# Bridging the translational gap: what can synaptopathies tell us about autism?

**DOI:** 10.3389/fnmol.2023.1191323

**Published:** 2023-06-27

**Authors:** Ciara J. Molloy, Jennifer Cooke, Nicholas J. F. Gatford, Alejandro Rivera-Olvera, Sahar Avazzadeh, Judith R. Homberg, Joanes Grandjean, Cathy Fernandes, Sanbing Shen, Eva Loth, Deepak P. Srivastava, Louise Gallagher

**Affiliations:** ^1^Department of Psychiatry, School of Medicine, Trinity College Dublin, Dublin, Ireland; ^2^Forensic and Neurodevelopmental Sciences, Institute of Psychiatry, Psychology and Neuroscience, King’s College London, London, United Kingdom; ^3^Kavli Institute for Nanoscience Discovery, Nuffield Department of Clinical Neurosciences, University of Oxford, Medical Sciences Division, Oxford, United Kingdom; ^4^Donders Institute for Brain, Cognition, and Behaviour, Radboud University Medical Centre, Nijmegen, Netherlands; ^5^Physiology and Cellular Physiology Research Laboratory, CÚRAM SFI Centre for Research in Medical Devices, School of Medicine, Human Biology Building, University of Galway, Galway, Ireland; ^6^Department of Medical Imaging, Radboud University Medical Centre, Nijmegen, Netherlands; ^7^Social, Genetic and Developmental Psychiatry Centre, Institute of Psychiatry, Psychology and Neuroscience, King’s College London, London, United Kingdom; ^8^MRC Centre for Neurodevelopmental Disorders, Institute of Psychiatry, Psychology and Neuroscience, King’s College London, London, United Kingdom; ^9^Regenerative Medicine Institute, School of Medicine, University of Galway, Galway, Ireland; ^10^FutureNeuro, The SFI Research Centre for Chronic and Rare Neurological Diseases, Royal College of Surgeons, Dublin, Ireland; ^11^Department of Basic and Clinical Neuroscience, Institute of Psychiatry, Psychology and Neuroscience, King’s College London, London, United Kingdom; ^12^The Hospital for SickKids, Toronto, ON, Canada; ^13^The Peter Gilgan Centre for Research and Learning, SickKids Research Institute, Toronto, ON, Canada; ^14^The Centre for Addiction and Mental Health, Toronto, ON, Canada; ^15^Department of Psychiatry, Temerty Faculty of Medicine, University of Toronto, Toronto, ON, Canada

**Keywords:** autism, NRXN1 deletion, Phelan-McDermid syndrome, SHANK3, synaptopathy, preclinical models, animal models, induced pluriopotent stem cells

## Abstract

Multiple molecular pathways and cellular processes have been implicated in the neurobiology of autism and other neurodevelopmental conditions. There is a current focus on synaptic gene conditions, or synaptopathies, which refer to clinical conditions associated with rare genetic variants disrupting genes involved in synaptic biology. Synaptopathies are commonly associated with autism and developmental delay and may be associated with a range of other neuropsychiatric outcomes. Altered synaptic biology is suggested by both preclinical and clinical studies in autism based on evidence of differences in early brain structural development and altered glutamatergic and GABAergic neurotransmission potentially perturbing excitatory and inhibitory balance. This review focusses on the NRXN-NLGN-SHANK pathway, which is implicated in the synaptic assembly, trans-synaptic signalling, and synaptic functioning. We provide an overview of the insights from preclinical molecular studies of the pathway. Concentrating on NRXN1 deletion and SHANK3 mutations, we discuss emerging understanding of cellular processes and electrophysiology from induced pluripotent stem cells (iPSC) models derived from individuals with synaptopathies, neuroimaging and behavioural findings in animal models of Nrxn1 and Shank3 synaptic gene conditions, and key findings regarding autism features, brain and behavioural phenotypes from human clinical studies of synaptopathies. The identification of molecular-based biomarkers from preclinical models aims to advance the development of targeted therapeutic treatments. However, it remains challenging to translate preclinical animal models and iPSC studies to interpret human brain development and autism features. We discuss the existing challenges in preclinical and clinical synaptopathy research, and potential solutions to align methodologies across preclinical and clinical research. Bridging the translational gap between preclinical and clinical studies will be necessary to understand biological mechanisms, to identify targeted therapies, and ultimately to progress towards personalised approaches for complex neurodevelopmental conditions such as autism.

## 1. Introduction

Autism spectrum condition (henceforth autism) is a prevalent neurodevelopmental condition associated with difficulties in communication and social interaction, restricted and repetitive behaviours, and sensory sensitivities (APA, 2013). Autism is highly heterogenous phenotypically and biologically, with multiple molecular pathways implicated. Autism is also highly heritable, with estimates of between 64 and 91% (Tick et al., 2016). A recent whole exome sequencing study identified 102 genes linked to autism, with separable functions mainly including gene expression regulation and neuronal communication, and few identified with cytoskeleton function (Satterstrom et al., 2020). An enrichment of genes encoding for proteins that directly regulate and/or localise to synapses, indicating synaptic function as one biological process that may be altered (Bourgeron, 2009; De Rubeis et al., 2014). This is supported by evidence from postmortem studies showing alterations in spine density morphogenesis and dendrite number (Raymond et al., 1995; Mukaetova-Ladinska et al., 2004; Hutsler and Zhang, 2010), as well as increased spine density in frontal, temporal and parietal lobes, and associations of spine density with cognitive function (Hutsler and Zhang, 2010). This may corroborate with grey matter volume differences reported in early development (Courchesne et al., 2001; Courchesne, 2004; Courchesne et al., 2011), as well as the altered connectivity hypothesis of autism (Geschwind and Levitt, 2007). Emerging evidence from both animal and human studies further suggest that an imbalance between excitatory and inhibitory (E/I) neurotransmission may be a common pathophysiological mechanism and hence a treatment target in autism (Yizhar et al., 2011; Gaetz et al., 2014; Rojas et al., 2015; Port et al., 2016; Horder et al., 2018; Bruining et al., 2020), with genetic evidence for multiple direct and indirect pathways to E/I imbalance (Satterstrom et al., 2020).

Synaptopathies refer to altered synaptic function in the brain linked to neurodevelopmental or neurological conditions (Brose et al., 2010). From a gene-based perspective, synaptopathies are conditions in which rare genetic variants impact genes directly expressed at the synapse, and may affect synaptic development, maintenance or signaling. Importantly, this definition does not encompass genes that indirectly impact synaptic function and may therefore represent an oversimplified approach to understanding synaptic function in neurodevelopmental conditions such as autism. For example, one study suggested that autism-related regulatory expression genes may not regulate autism-related neuronal communication genes, however, they may still impact synaptic function by altering neuronal numbers (Satterstrom et al., 2020). Further, as autism is highly complex with variable brain and trait developmental trajectories from childhood to adulthood (Riglin et al., 2021; Georgiades et al., 2022), a mechanistic approach to translational research using synaptopathies will likely be informative for some autistic individuals with altered synaptic function.

In this review we focus on two molecules from the NRXN-NLGN-SHANK pathway, namely NRXN1 and SHANK3, where there have been investigations across preclinical and clinical research. Further the NRXN-NLGN-SHANK pathway has been associated with E/I balance (Bourgeron, 2009), and NRXN1 and SHANK3 deletions have both been implicated in autism. For example, copy number variants (CNV) in *NRXN1* are reported to be present in about 0.4% of autistic people, and *SHANK3* in 0.05–0.7%, which increases to 2% in autistic people with moderate to profound intellectual disability (Devlin and Scherer, 2012; Leblond et al., 2014).

Synaptopathy focused research aims to uncover understanding of neurobiological mechanisms linked to autism at a molecular level, and how they contribute to clinical and behavioural phenotypes (Südhof, 2008). Preclinical and clinical studies are imperative to determine the functional impact of alterations to synaptic genes, and this mechanistic research approach has potential to increase discovery and development of treatment targets. For example, the availability of personalised prescreening drug platforms using patient derived induced pluripotent stem cell (iPSC) models can advance care for ultra-rare disorders (Sequiera et al., 2022). In the context of rare genetic conditions associated with autism, there is potential for personalised approaches to treatment that can ultimately improve quality of life and alleviate some traits that may be challenging for autistic people and their families to manage. This may also be valuable for those that may not respond to behavioural interventions alone. Importantly, translational research is focused on enhancing healthcare treatment choices for people who may wish to avail of them.

Drug discovery and success rates in clinical trials are extremely low, despite the vast amount of biomedical and translational research being implemented. Factors that may impede the effective translational of preclinical research to therapy include lack of communication between clinical and preclinical researchers, poor understanding of clinical relevance, efficacy issues, difficulties with reproducibility, species differences, as well as funding access and the high costs of developing and testing drugs at preclinical and clinical trial phases (Seyhan, 2019). Although research protocols and regulations have improved over time, there is still a high failure of therapeutics initially tested in preclinical animal models when tested in humans. There is a gap in our knowledge that needs to be addressed at both the preclinical and clinical level. The objective of this review is first to give a brief overview of the NRXN-NLGN-SHANK3 pathway and then to highlight key research findings in clinical and preclinical synaptopathy studies to date including both murine models and human iPSC research in NRXN1 and SHANK3 models. We will then examine the overlaps and differences in findings across clinical and preclinical research fields, identify the gaps in synaptopathy translational research, discuss the existing challenges, and finally establish potential solutions to align methodologies across preclinical and clinical research, with a specific focus on research in NRXN1 and SHANK3.

## 2. Overview of NRXN-NLGN-SHANK pathway

### 2.1. Neurexins

Neurexins (NRXNs) are a large family of cell adhesion molecules which facilitate trans-synaptic adhesion, synapse maturation and the establishment of synaptic identity (Südhof, 2017). These functions are mediated via multiple trans-synaptic interacting partners including neuroligins (NLGNs; Südhof, 2017). The genes encoding NRXNs consist of *NRXN1*, *NRXN2*, and *NRXN3* regulated by three different promoters for each gene resulting in alpha and beta, variants of each, and gamma variants of *NRXN1* (Tabuchi and Südhof, 2002; Sterky et al., 2017). Each of these isoforms are highly alternatively spliced in developing and mature neurons (Lukacsovich et al., 2019). Every NRXN protein translated from a spliced transcript can be further alternatively spliced to include or exclude various subdomains within each NRXN protein (Südhof, 2017). Ultimately, such varied alternative splicing at gene, transcript, and protein levels leads to one of the largest and most diverse splice isoform libraries in mammalian neurons (Flaherty et al., 2019). Efforts to characterise this library across the mammalian brain have underestimated this library’s scale. For example, long-read mRNA sequencing of NRXN1a and NRXN3a in one brain region at one time point revealed over 1,000 different splice isoforms (Treutlein et al., 2014; Flaherty et al., 2019). This suggests NRXNs have highly diverse expression patterns throughout the brain which differs spatially based on cell type, brain region or cortical layer, and temporally throughout neurodevelopment (Fuccillo et al., 2015; Harkin et al., 2017; Uchigashima et al., 2019).

At synapses, NRXNs are believed to contribute to the molecular code of synapse identity in conjunction with their extensive catalogue of trans-synaptic binding partners (Südhof, 2017). NRXNs are found at both excitatory and inhibitory synapses. Given the degree of alternative splicing, it is perhaps unsurprising that NRXNs operate in an exceedingly complex manner at synapses. For example, NRXN1 was recently shown to form nanoclusters in synaptic active zones (Trotter et al., 2019; Han et al., 2022). As for Nrxn2, a subset of splice variants have been shown to reduce synapse numbers and release probability (Lin et al., 2023). Combined with the previously hypothesised molecular code of synaptic identity, this suggests a further layer of convolution in understanding the role NRXNs play at synapses at the nanoscopic level. Moreover, NRXN1 has been shown to have a rich diversity of binding partners (Südhof, 2017). These include the family of NLGNs (Craig and Kang, 2007), as well as other proteins such as Leucine-rich-repeat transmembrane neuronal proteins (LRRTMs), GABA-A-receptors, cerebellins, and latrophilins (Südhof, 2017). Such molecular intricacy suggests either substantial functional redundancy or, more likely, a deeply entangled system of which current research has barely scratched the surface.

Due to this molecular complexity, NRXN mutations are well poised to contribute to E/I imbalance. As NRXNs interact with a diverse library of synaptic binding partners in an isoform dependent manner, mutations in NRXNs may induce synaptic mismatches at the individual synapse level (Dai et al., 2019). For example, alternative splicing of NRXN1 or NRXN3 at splice site 4 (NRXN1^SS4+^/NRXN3^SS4+^) mediates their binding to cerebellin-2 at synapses. This binding specificity in turn differentially regulates synaptic NMDA/AMPA receptors through the control of ionotropic glutamate delta receptors, GluD1 and GluD2 (Dai et al., 2021, 2022). Autism-associated mutations in this highly specific system will likely cause mismatches in the molecular code underlying synapse specificity during synapse specification and maturation. Therefore, potentially contributing to synaptopathies in a compounding manner as such mismatches are likely to be repeated within and between specific brain regions throughout neurodevelopment. The emerging dynamics of NRXN isoforms also likely explains why NRXN mutations have been linked to disparate neurological and neurodevelopmental conditions (Cuttler et al., 2021). Critically, the tools required to assess the extent of spatial and temporal NRXN isoform diversity globally have either yet to be developed or to be applied to NRXNs. A combined short-read amplicon sequencing and long-read isoform sequencing approach in human model systems, akin to Flaherty et al. (2019) could provide insights into how NRXN isoforms operate spatio-temporally in the healthy brain and how they potentially contribute to E/I imbalance in autism and other neurodevelopmental conditions (Flaherty et al., 2019).

### 2.2. Neuroligins

Neuroligins (NLGNs) are a much smaller family of autism-associated post-synaptic cell adhesion molecules which facilitate trans-synaptic adhesion, synapse maturation and the establishment of synaptic identity in an activity-dependent manner (Varoqueaux et al., 2006; Chubykin et al., 2007; Südhof, 2008). These synaptic adhesion proteins were the first to be identified as binding partners for NRXNs (Craig and Kang, 2007). NLGN1 and NLGN2 preferentially localise at excitatory glutamatergic and inhibitory GABAergic synapses, respectively (Graf et al., 2004). NLGN3 is present on both synapse subtypes but operates particularly at glutamatergic synapses to nanocluster AMPA receptors (Budreck and Scheiffele, 2007; Han et al., 2022). NLGN3 R451C mutations have been shown to enhance excitatory synaptic transmission (Wang L. et al., 2022). NLGN4 has been confirmed on excitatory cortical synapses and inhibitory glycinergic retinal neuron synapses (Hoon et al., 2011; Marro et al., 2019). NLGN3 and NLGN4X have also recently been shown to mediate neuritogenesis at growth cones during earlier stages of neurodevelopment (Gatford et al., 2022). NLGNs typically homodimerize at the cell membrane but evidence suggests NLGNs also heterodimerize in an isoform specific manner. NLGN1/NLGN3, NLGN1/NLGN2, and NLGN2/NLGN3 heterodimers have been demonstrated *in vitro* (Poulopoulos et al., 2012; Bemben et al., 2018). Furthermore, autism-associated mutations in *NLGN* interfere with this dimerization in an isoform specific manner (Poulopoulos et al., 2012). This small but specific isoform diversity is currently believed to contribute to the molecular code of synapse identity during neurodevelopment (Südhof, 2017). It is therefore plausible that autism-associated mutations in the NLGN family, although rarer in occurrence, ultimately contribute to miswiring and E/I imbalance; a significant component of multiple neurodevelopmental conditions.

### 2.3. SHANKs

SH3 and multiple ankyrin repeat domain proteins (SHANKs) are a small family of autism-associated post-synaptic scaffolding molecules which organise macromolecular protein complexes and receptors at the post-synaptic density of glutamatergic neurons (Naisbitt et al., 1999; Tu et al., 1999; Monteiro and Feng, 2017). The *SHANK* genes encode three Shank proteins (Shank1, Shank2, Shank3). Each protein also exhibits a handful of isoforms with specific domains removed, e.g., Shank2a is missing the Ankyrin domain, Shank2b is missing the Ankyrin and SH3 domains, and Shank3b is missing the SAM domain (Wang et al., 2011; Leblond et al., 2012). *SHANKs* degree of alternative splicing does not begin to approach the range of NRXN splice isoforms but more closely resembles the smaller library of NLGN isoforms. Shanks also exhibit differential expression across the brain. Shank1 and Shank2 are primarily expressed in the cortex, thalamus, hippocampus, dentate gyrus, and cerebellum (Böckers et al., 2004; Peça et al., 2011). Shank3, arguably the most well-characterised Shank, exhibits spatial, temporal, and isoform-dependent differential expression subcellularly and across neurodevelopment, e.g., Shank3a and Shank3e are enriched in the cytoplasm of striatal neurons, Shank3c and Shank3d are enriched in the cerebellum, Shank3b is highly expressed in the nuclei hippocampal neurons (Wang et al., 2014; Mei et al., 2016; Monteiro and Feng, 2017). This suggests multiple context-dependent functions for Shank3 rather than a generalised master regulator of synaptic structure. Due to its role as a structural synaptic scaffold for AMPA/NMDA receptors, Shank3 research has principally focused on synaptic dysfunction as the primary driver of pathology in autism in that Shank3 mutations likely interfere with the balance of these receptors. Indeed, multiple studies in hiPSC-neurons and rodent models of autism have shown a hyperexcitability phenotype, strongly implicating Shank3 in E/I imbalance (Shcheglovitov et al., 2013; Yi et al., 2016; Chiola et al., 2021). Translating such findings from *in vitro* to *in vivo* or vice versa has been frequently demonstrated but translating from bench to bedside has yet to prove fruitful.

## 3. Preclinical models of synaptopathies

### 3.1. NRXN1 iPSC research findings

IPSCs have emerged as an attractive platform for disease modelling, recapitulating human clinical pathophysiology. An attractive aspect of differentiating iPSCs towards a neuronal fate using a directed differentiation approach, is that iPSCs recapitulate the hall marks of human neurodevelopment (Kelava and Lancaster, 2016). For example, differentiation of iPSCs from autistic individuals with *NRXN1* deletions towards a cortical fate, were recently shown to exhibit impaired neurogenesis and altered neural rosette formation compared to cortical iPSC-neurons from neurotypical individuals (Adhya et al., 2021b). This was concurrent with an alteration in the number of excitatory and inhibitory progenitor cells being generated (Adhya et al., 2021b). Additionally, single cell analysis of autism iPSC-neurons with a bi-allelic NRXN1-α deletion revealed skewed fate choice in neural progenitors towards astroglial fate rather than primarily cortical neuron fate (Lam et al., 2019).

The functional impact of heterozygous deletion of NRXN1 has been investigated in human-derived isogenic embryonic stem cells (ESC) and iPSCs from individuals with schizophrenia and autism (Pak et al., 2015, 2021; Avazzadeh et al., 2019, 2021). The engineering of heterozygous schizophrenia-associated NRXN1 mutations induced electrophysiological impairment in neurons generated through the expression of neurogenin-2 (Pak et al., 2015). These electrophysiological changes occurred with an upregulation of NRXN1 intracellular binding partner CASK (Pak et al., 2015, 2021). These alterations include reduced neurotransmitter probability release and excitatory synaptic transmission with no significant change in overall neuronal development. Importantly, these phenotypes were absent in the mouse ESC model of the same NRXN1 heterozygous deletion, suggesting potential species difference and indicating the potential of using iPSCs to recapitulate the precise phenotype (Pak et al., 2015, 2021).

Directed differentiation of iPSCs from autistic individuals, resulted in the generation of cortical neurons with increased calcium signalling and neuronal excitability (Avazzadeh et al., 2019, 2021). These findings are, at the surface, in direct conflict with those generated by Pak et al. (2015, 2021). However, there are several important methodological differences to note between these studies. Firstly, Pak et al. used a forward programing method, with the overexpression of neurogenin-2, to generate a pure population of glutamatergic neurons. Of note, this approach results in an abridged neurodevelopmental period and results in a heterogenous population of glutamatergic neurons that are not specific to forebrain neurons (Spitzer, 1994). Previous studies have demonstrated that the neurodevelopmental period is key for the appearance of cellular and molecular phenotypes associated with the directed differentiation of autism-iPSCs (Schafer et al., 2019). In addition, Pak et al. (2015, 2021) generated both isogenic ES/iPSC lines, targeting exon 19, as well as iPSCs from schizophrenic individuals with heterozygous exonic deletions within NRXN1 (Pak et al., 2015, 2021). Conversely, Avazzadeh et al. (2019, 2021) generated iPSCs from autistic individuals with heterozygous deletions in NRXN1 using a dual SMAD inhibition differentiation protocol (Avazzadeh et al., 2019, 2021). This resulted in the generation of a combination of neuronal cell types, which differed from those observed in Pak et al. studies (Pak et al., 2015, 2021). In addition, the nature of the deletions could impact the splicing of NRXN1, similar to what has been previously shown (Flaherty et al., 2019). Moreover, this presents a central key question as to whether the distinct phenotypic outcomes are due to discrepancies in neurodevelopmental condition background (Trubetskoy et al., 2022). It is possible that other variants within the genome, associated with either increased likelihood for schizophrenia vs. autism may also contribute to phenotypic differences seen between these studies. Genes related to synaptic function are overrepresented among the common variants associated with schizophrenia. Interestingly, Avazzadeh et al. (2019) also reported that some of their autism cohorts also had a history of seizures and epilepsy, reinforcing the importance of a clear clinical diagnosis and its relative to phenotypic observations in *in-vitro* modelling of neurodevelopmental conditions (Avazzadeh et al., 2019). Alternate splicing is well conserved among neurexin genes, suggesting their important functional roles. However, while wild-type isoforms are generally conserved, mutant isoforms can be more diverse with over 30 isoforms identified in NRXN1+/− cases with schizophrenia (Flaherty et al., 2019). In the Psychiatric Genomics Consortium dataset, up to 23% of the heterozygous deletions in NRXN1 have a higher tendency to generate mutant isoforms, increasing their capability of exacerbating the disease phenotypes (Flaherty et al., 2019). iPSC-derived NRXN1+/− neurons from individuals with schizophrenia showed a reduction in neuronal activity that ameliorated with overexpression of the wild-type NRXN1α. Additionally, the presence of mutant isoforms in neurotypical iPSC neurons also exhibited reduced neuronal activity, elucidating the fact that the phenotypic impact of NRXN1+/− mutations can occur through both reduction of NRXN1α isoform and the presence of mutant alleles (Flaherty et al., 2019). This shows that the presence of NRXN1α deletion may have a dominant negative activity, however, future studies are needed to explore how NRXN1 deletions perturb the splicing profile, leading to alteration in neuronal activity.

### 3.2. SHANK3 iPSC research findings

PMD iPSC-derived forebrain neurons exhibit impaired excitatory synaptic transmission concordant with reduced glutamate receptor expression and reduced synapse number directly caused by the loss of Shank3 (Shcheglovitov et al., 2013). These phenotypes were rescued by restoring Shank3 levels to baseline through lentiviral expression of Shank3 or by stimulating Shank3 expression through insulin-like growth factor 1 treatment. Furthering the link between SHANK3 mutation and synaptic deficits, cortical iPSC-neurons with hemizygous SHANK3 deletions transplanted into healthy mouse prefrontal cortex exhibited impaired AMPA-mediated excitatory synaptic transmission concurrent with significant reductions in excitatory synapse number, dendritic spines, and dendritic outgrowth (Chiola et al., 2021); findings that are also partly recapitulated in iPSC-derived telencephalic organoids with engineered SHANK3 hemizygosity (Wang Y. et al., 2022). Moreover, SHANK3 conditional knock-out (cKO) in human embryonic stem cell-derived cortical neurons leads to a hyperpolarisation channelopathy phenotype dependent on Shank3 interacting with hyperpolarization-activated cyclic nucleotide-gated channels (Yi et al., 2016). Shank3-cKO cortical neurons also exhibited decreased dendritic outgrowth; although this may be a cell-type specific phenotype as the opposite effect was shown in Shank3-KO placodal neurons (Kathuria et al., 2018). Combined, these studies indicate that the contribution of Shank3 to the E/I imbalance in autism stems from its role as a scaffold for AMPA/NMDA receptors at synapses. Mutations in Shank3 interfere with the balance of these receptors and often lead to a neuronal hyperexcitability phenotype. This phenotype also extends to the circuitry level.

### 3.3. Nrxn1 animal model behavioural and brain phenotypes

Initial studies using a mouse model for the human NRXN1 copy number variation (CNV) mutation, the Nrxn1α mouse knockout maintained on a mixed genetic background (C57BL6/SV129), found significant deficits in prepulse inhibition, a measure of sensorimotor gating, increased repetitive self-grooming behaviour, which has been proposed to model repetitive behavioural traits associated with autism, and impaired nest building behaviours that have been associated with altered social behaviour (Etherton et al., 2009). Phenotypes were also observed in Nrxn1α heterozygous mice, including increased responsiveness to novelty and accelerated habituation to novel environments in male Nrxn1α heterozygous mice (Laarakker et al., 2012), and deficits in social memory in both male and female Nrxn1α heterozygous mice (Dachtler et al., 2015). These studies were all conducted in Nrxn1α maintained on a mixed genetic background which can seriously confound observed phenotypes (Schalkwyk et al., 2007). Therefore, Grayton et al. (2013) generated Nrxn1α mutant mice on a pure genetic background (C57BL/6 J; Grayton et al., 2013). In this context, Nrxn1α knockout male mice displayed altered social approach, reduced social investigation, increased aggression and increased anxiety (Grayton et al., 2013). None of these studies tested whether there is a developmental trajectory for any of these behaviours or established a neurodevelopmental basis for the effect of the Nrxn1α deficiency. Armstrong et al. (2020) examined the effects of Nrxn1α deletion on behaviour across a range of developmental time points to determine whether potential abnormalities follow a developmental trajectory (Armstrong et al., 2020). Pups deficient in Nrxn1α (PND 2–12) emitted a reduced number of ultrasonic vocalizations combined with a restricted repertoire of calls indicative of a loss in complexity in vocal production and showed delays in reaching certain developmental milestones. Juvenile (PND30) and adult (PND65-67) male Nrxn1α knock-out mice exhibited social deficits and increased levels of aggression, but there were no differences in social behaviours or aggression in female Nrxn1α knock-out mice in this study. This confirmed previous findings (Grayton et al., 2013) and demonstrated that deletion of both Nrxn1α alleles does result in social and communication deficits that follow a sex-specific, developmental trajectory. A recent study by Xu et al. (2023) investigated the effect of an allelic series of Nrxn1 mouse models on behaviour, and they also found sex differences in behaviour with increased aggression in males and decreased passive interactions in female mice in a resident intruder test and altered circadian activities in both sexes following homozygous loss of Nrxn1α (Xu et al., 2023). Although Xu et al. (2023) refer to this passive interaction as an affiliative social behaviour, the resident intruder test is a test of agonistic not social behaviours (Koolhaas et al., 2013), so their finding cannot be interpreted as a change in social behaviour in female mice. Xu et al. (2023) also reported that both heterozygous and homozygous loss of Nrxn1α reduced the preference for social novelty in male mice and increased repetitive motor skills and motor coordination in both sexes. Taken together, these findings indicate the importance of Nrxn1α gene dosage and sex in regulating behavioural phenotypes in mouse.

Given the aggressive phenotypes observed in mice (Grayton et al., 2013), it has always been challenging to study more complex social behaviours in the murine Nrxn1α model. Rat models allow us to finely dissect the effects of Nrxn1α on social traits (Argue and McCarthy, 2015). A recent study using a rat model characterised the behavioural effects of Nrxn1α deficiency across early development in both sexes and identified similar phenotypes to those observed in earlier mouse studies (Kight et al., 2021). Both male and female Nrxn1α-deficient neonates displayed reduced ultrasonic vocalizations at postnatal day 4, 8 and 10 when separated from their mother. When juvenile (aged 27–30 days), sociability and social discrimination did not differ between mutant and wildtype rats, however a deficit in social play was observed in Nrxn1α-deficient male but not female rats. Further sex differences were observed in locomotor activity and object exploration, with male mutant juvenile rats showing increases in these behaviours compared to wildtype males but no differences seen in these behaviours in female mutant rats. Kight et al. (2021) explored more complex social behaviours and demonstrated that Nrxn1α deficiency resulted in reduced prosocial helping behaviour and learning deficits but interestingly, increased nurturing behaviour in both male and female juvenile mutant rats (Kight et al., 2021). Other studies focused on cognitive processing have suggested that there may be a core dysfunction in reward processing in Nrxn1α deficiency rats (Esclassan et al., 2015) and mice (Alabi et al., 2020). Less is known about the role of beta neurexins in neuronal function as these are expressed at reduced levels compare to alpha neurexins but conditional deletion of beta neurexins (1β, 2β and 3β) decreases release of neurotransmitters from excitatory synapses and impairs contextual fear memory when deleted in hippocampal CA1 neurons (Anderson et al., 2015).

Although some discrepancies exist, the majority of brain function studies in Nrxn1 animal models indicate E/I imbalance. Electrophysiology has revealed reduced spontaneous excitatory synaptic transmission and decreased excitatory synaptic strength in the CA1 region of the hippocampus of Nrxn1α knockout mice, but no change in inhibitory synaptic transmission (Etherton et al., 2009). This E/I imbalance was related to repetitive self-grooming behaviours, prepulse inhibition, sensorimotor gating and organised behaviour, but not social behaviours. However, in contrast one study reported no change in synaptic transmission when they examined cortical neurons cultured from both heterozygous and homozygous Nrxn1α knockout mice, which contradicted their findings in human ESC with heterozygous mutations (Pak et al., 2015).

Impaired excitatory transmission from the prefrontal cortex to the basal amygdala has also been reported in Nrxn1α knockout mice, along with impaired fear learning, suggesting altered amygdala fear circuit (Asede et al., 2020). In both Nrxn1α +/− and Nrxn1α−/− mice, decreased synaptic strength from dorsal prefrontal cortex to dorsal medial striatum has been shown, resulting from reduced glutamate release (Davatolhagh and Fuccillo, 2021). The imbalance in striatal circuit activity was suggested to be input specific. In a study using an analogue of positron emission tomography (PET) in Nrxn1α+/− mice, prefrontal cortex hypometabolism and dorsal raphe nucleus hypermetabolism were reported, which also correlated with enhanced novel discrimination of odors and longer latency to make correct choices, respectively, (Hughes et al., 2022). In NRXN1 knockout rats, altered cortico-thalamic-striatal circuit communication has also been reported, along with altered gamma band oscillations (Janz et al., 2022). Importantly, this has been linked with developmental delay in autism, and may reflect an E/I imbalance (Orekhova et al., 2007). Taken together, neurotransmission differences may be linked to altered neurotransmitter release and E/I balance, which may in turn impact behaviour and cognition.

Brain structural phenotypes of Nrxn1 knockout rats has also been explored using magnetic resonance imaging (MRI) methods such as diffusion tensor imaging (DTI) measures of white matter white matter microstructure and neurite orientation dispersion and density imaging (NODDI) measures of neurite density showing reduced microstructural integrity in the left capsule and right neocortex, but no difference in neurite density (Barnett et al., 2020). Overall, the presence of both structural and functional differences in brain development in Nrxn1 deletion animal models is indicated, which related to observed differences in behaviours in some cases. However, developmental trajectories are yet to be studied more comprehensively.

### 3.4. Shank3 animal model behavioural and brain phenotypes

The behavioural differences reported for *Shank3* mutant mice depend on the strains and experimental conditions (Delling and Boeckers, 2021). Importantly, a clear pattern of phenotype related to a specific Shank3 isoform has not been reported (Ferhat et al., 2023). In general, complete deletion models of SHANK3 lacking all protein isoforms by removing exons 4–22 enable the study of basic biological functions of SHANK3 by eliminating isoform compensation (Drapeau et al., 2018; Delling and Boeckers, 2021; Ferhat et al., 2023). While it has been argued that heterozygous SHANK3-deficient rodents better represent the haploinsufficiency of PMD, behavioural phenotype studies are less replicated than homozygous SHANK3-deficient rodents (Delling and Boeckers, 2021). Heterozygous SHANK3-deficient rodents are also reported to show a less severe phenotype (Delling and Boeckers, 2021).

The presence of repetitive behaviours, a core feature of autism and PMD, is the most robust phenotype observed in the Shank3-KO murine models of autism. Excessive self-grooming behaviours have been consistently reported in various rat and mouse lines carrying Shank3 mutations, with skin lesions and self-injurious behaviour reported in Shank3B knockout mice (Peça et al., 2011; Wang et al., 2011; Yoo et al., 2019; Matas et al., 2021; Ferhat et al., 2023). Shank3B knockout mice are thought to show a more severe behavioural phenotype, however, other studies have suggested milder grooming behaviours (Wang et al., 2011; Kabitzke et al., 2018).

Mouse models have been shown to also exhibit significant variations in other autism-like behaviour such as social communication and interaction. Reduced ultrasonic vocalisations are reported across different Shank3 models. As for other social behaviours findings are mixed. In complete knockout of Shank3 (exon 4–22) normal levels of social interest, but persistence in unsuccessful efforts to engage the social partner were found (Wang X. et al., 2016), whereas no differences in social recognition have been reported in two Shank3 mouse models that included a deletion of exons 13–16 of the PDZ domains and deletions of exons 4–9 of the Shank3 gene (Kabitzke et al., 2018). Another study also reported no difference in social preference or dyadic male–female social interaction (Drapeau et al., 2018). As rodent social behavior is highly influenced by experimental conditions, discrepancies across studies in social behaviours may be due to the variations in the different procedures used to evaluate social interaction (Drapeau et al., 2018, Delling and Boeckers, 2021, Ferhat et al., 2023).

Altered rearing behaviours, thought to reflect anxiety-like behaviour, have been observed in Shank3 mice models also (Peça et al., 2011; Kabitzke et al., 2018). For example, anxiety-like behaviour has been reported in Shank3B knockout mice, but not Shank3A knockout mice with a milder phenotype (Peça et al., 2011). Hypoactivity and reduced spontaneous locomotor activity have also been reported (Drapeau et al., 2018; Kabitzke et al., 2018), as has reduced motor learning (Drapeau et al., 2018) and gait abnormalities (Matas et al., 2021). Sleep patterns have also been examined, and Shank3 mouse models lacking exon 21 have abnormal sleep with earlier onset of diurnal/nocturnal sleep/wake distribution, less sleep overall throughout the lifespan, and take longer to fall sleep as adults (Ingiosi et al., 2019; Medina et al., 2022).

Studies have demonstrated brain phenotypes related to Shank3 indicating differences in brain functional and structural development, with some suggesting that disruption to Shank3 in specific brain regions may be linked to specific behavioural outcomes. For example, repetitive behaviours are frequently associated with anomalies of the striatum (Kalueff et al., 2016). In this regard, anatomical, circuit, and cellular abnormalities have been identified in the striatum of Shank3-KO mice, including larger volume, disruption of the cortico-basal ganglia indirect pathway, and aberrant basic cellular excitability of medium spiny neurons (Peixoto et al., 2016; Wang X. et al., 2016; Reim et al., 2017; Wang et al., 2017; Bey et al., 2018; Schoen et al., 2019; Jin et al., 2021). Additionally, tonic hyperactivity in the cortico-striatal-thalamic circuits has been associated with impaired Shank3/Homer1-mediated mGluR5 activity (Wang X. et al., 2016). Changes in the mGluR5 localisation have been also observed in the hippocampus of mice lacking Shank3 exon 21, together with a decreased NMDA/AMPA excitatory current ratio, suggesting an altered balance between excitation and inhibition (Kouser et al., 2013).

Several studies using different animal models of Shank3 deficiency have reported diverse changes in the synaptic function associated with shifts in the E/I balance. For instance, cortico-striatal hyperactivity has been observed in developing Shank3-KO mouse spiny projection neurons leading to cortico-striatal hyperconnectivity and thus E/I imbalance between cortical and striatal regions (Peixoto et al., 2016). However, mice lacking Shank3 exon 9 exhibited a reduction in the excitatory transmission in the hippocampal CA1, increased frequency of inhibitory synaptic events in pyramidal neurons, and decreased frequency of inhibitory synaptic events in medial prefrontal cortex layer 2/3 neurons (Lee et al., 2015). On the other hand, a mice line lacking Shank3 exon 13–16 displayed heightened sensory sensitivity linked to an increase in the spontaneous and stimulus-evoked activity of excitatory neurons in the somatosensory cortex together with a decrease in interneuron activity (Chen et al., 2020). Most recently, a study using a circuit selective mutation strategy to study Shank3 deficiency in the PFC-BLA circuit has shown altered neuronal morphology, increased post-synaptic excitatory activity, and reduced inhibitory activity. This cortico-amygdala hyperactivity led to impaired social interaction through temporal disruption of socially tuned neurons (Kim et al., 2022). Each of these mouse models of autism also exhibited significant variations in multiple autistic-like behaviours such as reduced ultrasonic vocalisations, altered rearing behaviours, and increased repetitive behaviours (Peça et al., 2011). The complex nature of the E/I imbalance phenotypes and autistic-like behaviours found in each of these studies suggests that the contribution of Shank3 to E/I imbalance in autism is sub-region and likely cell-type specific.

A translational study using proton magnetic resonance spectroscopy ([1H]MRS) in humans and six diverse autism rodent models, including Shank3, reported glutamatergic and GABAergic genetic links to autism traits (Horder et al., 2018). GABA levels were not altered in the striatum or prefrontal cortex in either the autistic adults or the Shank3 KO animal model. In autistic adults, glutamate concentration was reduced in the striatum of autistic adults but not the prefrontal cortex. Reduced glutamate concentration in the striatum correlated with measures of social interaction and communication. The reduction in striatal glutamate was only recapitulated in mice prenatally exposed to valproate, and in mice carrying Nlgn3 mutations, as well as a tendency toward reduction in striatal glutamate of the Shank3 KO mice. These changes were not observed in rodent autism models with other etiologies, suggesting that glutamate/GABA abnormalities in the corticostriatal circuitry may be a key pathological mechanism in autism-related synaptopathies (Horder et al., 2018).

Brain structure of Shank3 murine models has been examined using MRI, with volumetric analyses indicating increased striatum volume (Peça et al., 2011; Wang X. et al., 2016), and decreased whole brain volume (Ellegood et al., 2015; Schoen et al., 2019), as well as hippocampus (Schoen et al., 2019) and prefrontal cortex volume (Pagani et al., 2019). In addition, DTI identified alterations in white matter structural architecture in rats with Shank3 deficiency specifically in the external capsule (Golden et al., 2021). A translational cross-species approach has also been used to uncover white matter structural architectural differences that overlapped in people with PMD and a Shank3 mouse model. The study revealed differences in white matter structural differences in long fiber tracts across species, with alterations in frontal tracts including the uncinate fasciculus and the inferior fronto-occipital fasciculus as well as the corticostriatal pathway in PMD, and differences in the fronto-occipital association fiber tract and the tract connecting motor cortex to entorhinal cortex in Shank3 mouse model (Jesse et al., 2020). Overall, Shank3 murine model studies indicate differences in both brain structural and functional development, which impact behavioural and cognitive outcomes. The overlap in differences in cross-species studies suggest the potential to identify of cross-species biomarkers that warrant further study.

## 4. Human clinical synaptopathies

### 4.1. NRXN1 deletion clinical, behavioural and brain phenotypes

Mono-allelic or heterozygous deletions at 2p16.3 involving the *NRXN1* gene are associated with a range of neurodevelopmental and neuropsychiatric conditions, whereas homozygous or bi-allelic deletions and compound heterozygous deletions are known as Pitt-Hopkins-like syndrome, a rare autosomal recessive syndrome which presents as a more severe phenotype (Zweier et al., 2009; Harrison et al., 2011; Béna et al., 2013; Lowther et al., 2017; Cosemans et al., 2020). For this review we focus on heterozygous NRXN1 deletions.

NRXN1 deletions vary in the location and size of the deletion or genetic alteration. The most frequently occurring deletions are located at exon 1–5 are associated with a wide range in clinical phenotypes, whereas exon 6–24 deletions are associated with higher rate of *de novo* and a more severe neurodevelopmental and neuropsychiatric phenotype, mainly intellectual disability and schizophrenia (Cosemans et al., 2020). Exonic and intronic deletions have been described in clinical cohorts with NRXN1 deletions with the former reported to have an elevated likelihood for neurodevelopmental conditions, autism and intellectual disability (Lowther et al., 2017). Some studies suggest that intronic deletions are not clinically significant (Lowther et al., 2017), however, some disrupt one of the 7 canonical splice sites in *NRXN1* and theoretically may be more likely to be associated with clinical phenotypes but this has not been systematically evaluated. *NRXN1* is thought to be generally intolerant of missense variants, although very rare cases carrying these mutations have been reported. For example, missense variants predicted to be protein damaging impacting the LNS4 domain of *NRXN1α* isoform have been found in autism and schizophrenia (Ishizuka et al., 2020).

NRXN1 deletions may be inherited from a parent (who may or may not have a neurodevelopmental or neuropsychiatric condition) or arise *de novo.* Variable and incomplete penetrance is observed in association with NRXN1 deletions. Carrier parents are thought to be unaffected based on clinical reports (Dabell et al., 2013), but there are no studies that have systematically evaluated parental carrier phenotypes. Heterozygous exonic deletions are observed in the general population with an estimated frequency of about 0.02–0.028% (Ching et al., 2010; Kirov et al., 2014; Rees et al., 2014), compared to 0.18–0.4% in autism/intellectual disability and schizophrenia populations (Ching et al., 2010; Devlin and Scherer, 2012; Rees et al., 2014; Kirov, 2015).

In clinical cohort studies, moderate to severe intellectual disability (77–92%) and autism are most commonly reported (43–70%; Castronovo et al., 2020) and speech and language delay also occurs in a high percentage of carriers of either exonic or intronic deletions (Al Shehhi et al., 2019). Speech development is highly variable in children with NRXN1 deletions ranging from no difficulties to phonological delay and/or articulation disorder, or abnormal oromotor function and both expressive and receptive language delay is reported (Brignell et al., 2018). NRXN1 deletions are also associated with attention deficit hyperactivity disorder (ADHD), schizophrenia, anxiety, and Tourette syndrome (Béna et al., 2013; Dabell et al., 2013; Al Shehhi et al., 2019), and in some cases obsessive compulsive disorder, epilepsy, macrocephaly, congenital anomalies and hypotonia (Møller et al., 2013; Al Shehhi et al., 2019; Castronovo et al., 2020).

Given the highly heterogeneous clinical phenotype in NRXN1 deletion carriers, there is a strong need to understand the full range of genetic, biological and environmental factors that may influence clinical outcome. One clinical study investigated neurodevelopmental outcomes in NRXN1 deletion carriers along multiple neurodevelopmental-related CNV using parent-report questionnaire data. They reported increased autism and hyperactivity features, and poorer social functioning traits in children with NRXN1 deletions compared to neurotypical children (Chawner et al., 2019). Although autism is prevalent in people with NRXN1 deletions, related features such as sensory processing or sensory sensitivities have not yet been examined in-depth. Sleep problems are a common feature of autism and developmental disabilities. A recent study was the first to show sleep disturbances in NRXN1 deletion, specifically increased rates of insomnia and tiredness and fatigue (Chawner et al., 2023).

Neurocognitive and neuroimaging biomarker research in NRXN1 deletions is in its infancy (Cooke et al., 2022). To date, no neurocognitive eye-tracking studies, or EEG studies of sensory or cognitive processing have been completed, with reports only in relation to epilepsy (Møller et al., 2013). Similarly, no MRI studies of brain structure and function have been reported in NRXN1 deletion CNV carriers. Only one MRI study of intronic SNP variant of NRXN1 reported an enlargement of the temporal horns of lateral ventricles which associated with psychosis (Alliey-Rodriguez et al., 2019). Further research into brain and cognitive phenotypes in clinical is necessary to progress translational research.

### 4.2. SHANK3 deletion (Phelan-McDermid syndrome, PMD) clinical, behavioural and brain phenotypes

PMD is typically associated with deletions at 22q13.3 that can extend up to 9 Mb, affecting up to 90 genes incorporating the *SHANK3* gene (Harony-Nicolas et al., 2015). Genetic deletions typically occur *de novo,* are paternally derived, and have high penetrance. Cases of PMD resulting from point mutations in *SHANK3* have increased in recent years due to the prevalence of more large-scale sequencing studies (Sarasua et al., 2011; Soorya et al., 2013; De Rubeis et al., 2018). Autism traits have been strongly linked to haploinsufficiency of SHANK3 (including point mutations; Sarasua et al., 2011; Soorya et al., 2013; De Rubeis et al., 2018; Kolevzon et al., 2019). Larger deletions encompassing multiple genes have been linked to severity of developmental delay, presence of epilepsy, neonatal hypotonia, certain dysmorphic facial features, and delayed language development (Sarasua et al., 2011).

Neonatal hypotonia is the earliest reported indicator of PMD, with poor feeding, speech difficulties, and delayed motor milestones evident in the first years of life (Phelan and McDermid, 2011). As a child develops, absent or delayed speech, moderate to profound developmental delay, and autistic traits characterise the syndrome (Phelan et al., 1993; Phelan and McDermid, 2011; Soorya et al., 2013; Kolevzon et al., 2014). Autistic features are associated with the loss of at least one functional copy of SHANK3 (Durand et al., 2007; Kolevzon et al., 2014; Leblond et al., 2014). Approximately 70% of individuals with PMD receive a diagnosis of autism. Around 40% of individuals with PMD will also develop seizures, meaning that epilepsy is a growing clinical consideration of the syndrome (Phelan et al., 1993).

Sleep problems are also frequently reported feature in PMD, with 89% of individuals reported to have sleep disturbance in one study (Bro et al., 2017), Individuals with PMD are more likely to use sleep aids than their unaffected siblings (Smith-Hicks et al., 2021). The same study found that the severity of sleep disturbances in PMD increased above the age of 11 years, which may relate to the physiological changes associated with puberty, and is consistent with the literature in other developmental disabilities, but not idiopathic autism, where sleep problems are not predicted by age or ability level (Reynolds and Malow, 2011; Smith-Hicks et al., 2021).

Other reported physical features of PMD include minor dysmorphic facial features, such as a wide brow, deep-set eyes, puffy eyelids, a bulbous nose, and long thick eyelashes (Phelan and McDermid, 2011). Sensory stimulating behaviours, such as chewing or mouthing objects (other than food), and reduced pain perception are also common (Phelan and McDermid, 2011; Vogels et al., 2021). There is some emerging evidence for a syndrome-specific sensory reactivity profile in PMD, which has been linked to the haploinsufficiency of *SHANK3* (Tavassoli et al., 2021). However, reported frequencies of sensory seeking behaviours in PMD are mixed, indicating that further investigation is warranted (Tavassoli et al., 2021; Lyons-Warren et al., 2022).

Loss of skills and/or psychiatric symptoms have been reported in adolescence and/or early adulthood, typically following an acute life event, such as a seizure, an infection, or a change in environment, i.e., moving into residential care (Kolevzon et al., 2019; Vogels et al., 2021). Presentations of periodic catatonia have also been described, along with certain symptom presentations that are consistent with bipolar disorder (irritability and mood dysregulation) or psychosis (paranoid delusions or hallucinations; Kolevzon et al., 2019). Importantly, there is considerable uncertainty in relation to reliable diagnosis of psychiatric conditions in moderate to profound intellectual disability and therefore findings in this area should be considered with caution (Vogels et al., 2021).

One study to date has examined attentional engagement and recognition memory in PMD using eye-tracking methodology, and found no discrimination between the viewing of social and non-social stimuli and a decay in recognition memory for PMD participants versus idiopathic autistic and neurotypical participants (Guillory et al., 2021). Findings point to the possibility of a dissociable pattern of attentional engagement and memory in PMD that stands apart from idiopathic autism (Guillory et al., 2021).

An electroencephalography (EEG) study, investigating neural signatures of PMD, have shown reduced latency and amplitude of Visual Evoked Potentials (VEPs) in PMD compared to neurotypical development (Siper et al., 2022). VEPs are thought to reflect excitatory and inhibitory neural dynamics within the cortex and reduction may reflect disruption to neural plasticity. Conversely, neural response (amplitude), latency, and habituation to simple auditory stimuli were found to be largely unaffected in PMD (Isenstein et al., 2022). However, early auditory processing differences in PMD seems to be related to the size and extent of genetic deletion (Isenstein et al., 2022). Another study showed PMD specific differences in Phase Amplitude Coupling, which is coupling of slow-wave phase with fast-wave amplitude oscillations, thought to reflect dynamic coordination of brain neural activity. The strength of Phase Amplitude Coupling was associated with autistic features, such as insistence on sameness/resisting change, ritualistic and compulsive behaviours (Mariscal et al., 2021).

Structural MRI studies indicate reduced basal ganglia volume in PMD (Srivastava et al., 2019), and mixed findings on cerebellum abnormalities (Aldinger et al., 2013; Srivastava et al., 2019). Noteworthy, both studies included small sample sizes. Arachnoid cysts have been reported to be present in about 15% of individuals with PMD (Phelan et al., 1993). In regards white matter, corpus callosum hypoplasia and general white matter thinning has been reported (Philippe et al., 2008).

One study has examined functional activity during a task measuring communicative and non-communicative sounds, and a PMD group showed increased activity for communicative sounds in the right superior temporal gyrus, whereas autism and neurotypical comparison groups did not (Wang A. T. et al., 2016). Both the left and right superior temporal gyrus are involved in speech processing (Kennedy-Higgins et al., 2020), the left superior temporal gyrus is thought to have a clearer role as aphasia is observed with damage to the left superior temporal gyrus but not the right (Luthra, 2021; Acharya and Wroten, 2022). However, it has been suggested that the left superior temporal gyrus is involved in category structure of speech sounds, while the right superior temporal gyrus may be involved in processing perceptual information about the voice (Luthra, 2021). While in neurotypical cohorts superior temporal gyrus activity is usually bilateral for communicative sounds, the differences in neural activity in PMD may reflect differences in language processing and communication compared to both neurotypical and autistic cohorts (Wang A. T. et al., 2016). Clinical phenotypes are well characterized in PMD, and further research to replicate and reproduce brain phenotypes is necessary to identify PMD brain markers.

## 5. Discussion

### 5.1. Parallels and inconsistencies across preclinical and clinical studies

**Figure 1:**
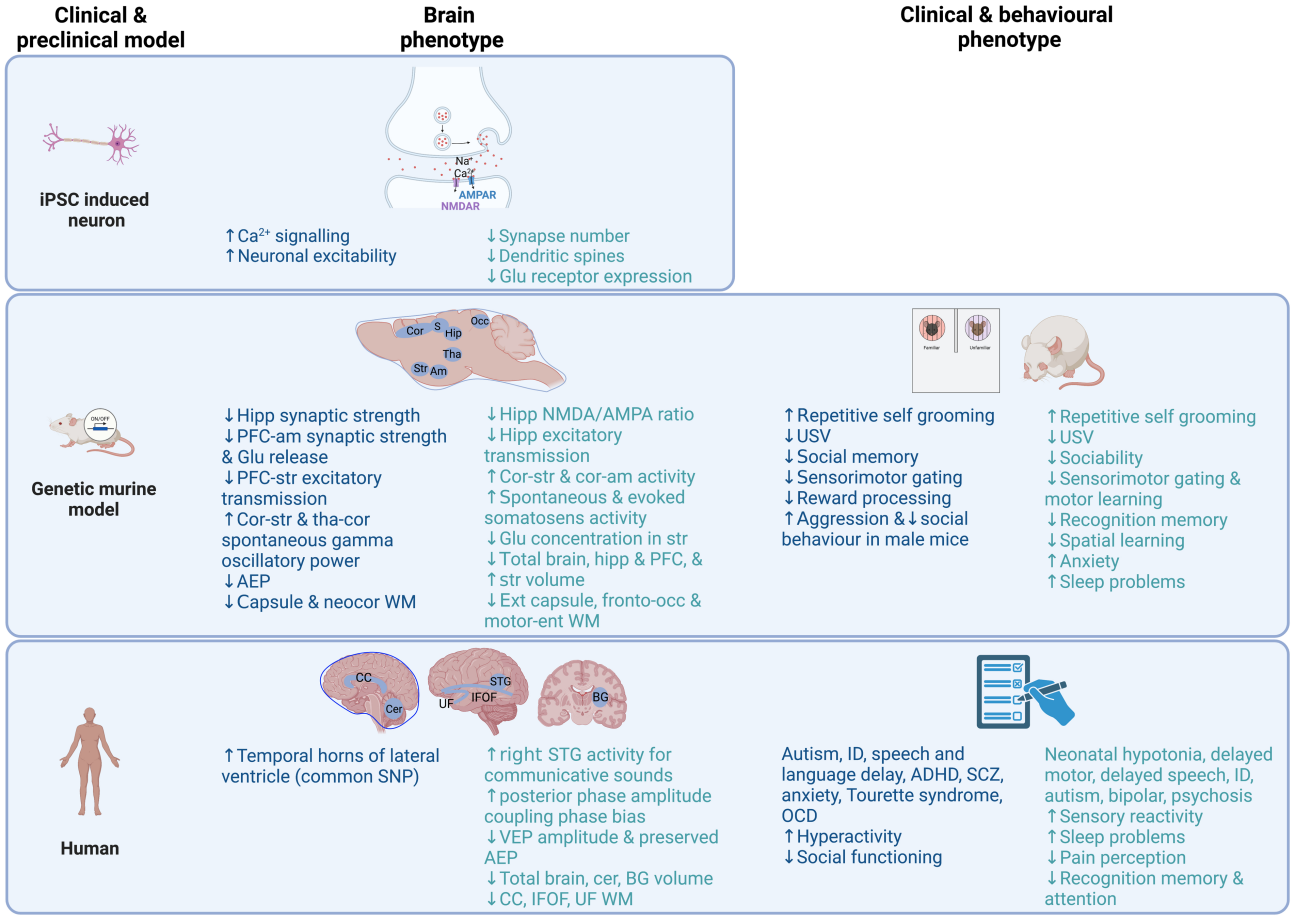
Overview of brain and behavioural phenotypes reported in clinical and preclinical model research studies of NRXN1/Nrxn1 deletion (Blue) and PMD/Shank3 (Green) to date. Noteworthy, many of the findings shown are based on single studies, therefore should be interpreted as preliminary findings, with future replication needed. Created with BioRender.com. ADHD, attention deficit hyperactivity disorder; AEP, auditory evoked potential; Am, amygdala; BG, basal ganglia; Ca2+, calcium; CC, corpus callosum; Cer, cerebellum; Cor, cortex; Ent, entorhinal; Glu, glutamate; Hipp, hippocampus; ID, intellectual disability; IFOF, inferior fronto-occipital fasciculus; Occ, occipital; OCD, obsessive compulsive disorder; PFC, prefrontal cortex; SCZ, schizophrenia; STG, superior temporal gyrus, Str, striatum; Tha, thalamus; UF, uncinate fasiculus; USV, ultrasonic vocalisations; VEP, visual evoked potential; WM, white matter.

The clinical, behavioural and brain phenotypes in iPSC, murine model and human clinical studies reviewed here are summarized in Figure 1 and Table 1 for NRXN1/Nrxn1 and Table 2 for PMD/Shank3. Behaviourally, Nrxn1 and Shank3 animal models display autism-related behaviours that are consistent with observations in clinical studies. These behaviours include reduced social communication and interaction, repetitive grooming, which is thought to parallel repetitive behaviours observed in autism and reduced ultrasonic vocalizations early in development. Motor learning and gait abnormalities are reported in Shank3 animal models, albeit to a lesser degree in female mouse models. Reduced sleep across the lifespan in Shank3 murine model parallels sleep disturbance in human PMD and autism studies.

**Table 1:** Summary of preclinical and clinical Nrxn1/NRXN1 deletion studies characterising behavioural and brain phenotypes.

	iPSC Model	Murine Model	Human
Clinical/behavioural phenotype	N/A	Male and female mice: ↑ Repetitive self -grooming behaviors (Etherton et al., 2009) ↓ Communication complexity in early development (ultrasonic vocalization and restricted repertoire of calls; Armstrong et al., 2020) ↓ Social memory (Dachtler et al., 2015) ↓ Sensorimotor gating (Etherton et al., 2009) ↓ Reward processing (Alabi et al., 2020) Male mice only: ↑ Responsiveness to novelty (Laarakker et al., 2012) ↑ Aggression and anxiety (Grayton et al., 2013) ↓ Social behaviour (Grayton et al., 2013) Male and female rats: ↑ Nurturing behaviour (Kight et al., 2021) ↓ Ultrasonic vocalization in early development (Kight et al., 2021) ↓ Prosocial helping (Kight et al., 2021) ↓ Learning (Kight et al., 2021) ↓ Reward processing (Esclassan et al., 2015) Male rats only: ↓ Social play (Kight et al., 2021) ↑ Locomotor activity and object exploration (Kight et al., 2021)	Clinical diagnosis most reported: Autism, intellectual disability and speech and language delay (Lowther et al., 2017; Al Shehhi et al., 2019; Castronovo et al., 2020) Other conditions: ADHD, schizophrenia, anxiety, Tourette syndrome, obsessive compulsive disorder (Béna et al., 2013; Dabell et al., 2013; Al Shehhi et al., 2019; Castronovo et al., 2020) Some reports of motor delay and hypotonia (Al Shehhi et al., 2019) ↑ Autism traits (Chawner et al., 2019) ↓ Social functioning(Chawner et al., 2019) ↑ Hyperactivity (Chawner et al., 2019) ↑ Sleep disturbances in some (Chawner et al., 2023)
Brain phenotype	↑ Calcium signalling in autism (Avazzadeh et al., 2019) ↑ Neuronal excitability in autism (Avazzadeh et al., 2021) ↓ Neuronal activity in schizophrenia (Pak et al., 2015) Upregulation of interacellular CASK protein in schizophrenia (Pak et al., 2015, 2021)	Mice: ↓ Hippocampal excitatory synaptic strength and no change in inhibitory synaptic transmission (Etherton et al., 2009) No change in synaptic transmission in cortical neurons (Pak et al., 2015) ↓ Glutamate release and synaptic strength from dorsal prefrontal cortex to dorsal medial striatum (Davatolhagh and Fuccillo, 2021) ↓ Excitatory transmission from dorsal medial prefrontal cortex to basal amygdala (Asede et al., 2020) PET analogue: Medial prefrontal cortex hypometabolism and dorsal raphé nucleus hypermetabolism (Hughes et al., 2022) Rats: ↑ Spontaneous gamma band oscillatory power and coherence in cortico-triatal and thalamocortical circuits (Janz et al., 2022) ↓ Auditory-evoked oscillations and evoked-potentials (Janz et al., 2022) DTI and NODDI: ↓white matter integrity in the left capsule and right neocortex, and no difference in neurite density (Barnett et al., 2020)	Epilepsy (Møller et al., 2013) MRI: ↑ temporal horns of lateral ventricles in common SNP (Alliey-Rodriguez et al., 2019) No MRI studies in CNV carriers

**Table 2:** Summary of preclinical and clinical Shank3/PMD studies characterising behavioural and brain phenotypes.

	iPSC	Murine model	Human
Clinical/behavioural phenotype	N/A	↑ Repetitive self- grooming (Wang et al., 2011; Yoo et al., 2019; Matas et al., 2021; Ferhat et al., 2023) ↓ Ultrasonic vocalisations (Peça et al., 2011) ↑Persistence in unsuccessful social partner engagement (Wang X. et al., 2016) ↓Sociability and interaction (Peça et al., 2011; Orefice et al., 2019), and no difference in social preference (Drapeau et al., 2018) or social recognition (Kabitzke et al., 2018) ↓Investigatory behaviour (Wang X. et al., 2016) Avoidance to novelty (Wang X. et al., 2016) ↑Anxiety (Wang X. et al., 2016; Bey et al., 2018; Drapeau et al., 2018) ↓ Sensory motor gating and ↓motor learning (Bey et al., 2018; Drapeau et al., 2018; Yoo et al., 2019) ↓ Recognition memory (Wang et al., 2011) ↓Spatial learning (Wang X. et al., 2016, Drapeau et al., 2018) ↓Striatal-dependent instrumental learning (Wang X. et al., 2016) Sleep problems (Ingiosi et al., 2019, Medina et al., 2022)	Clinical diagnosis: Neonatal hypotonia, delayed motor milestones, delayed or absent speech, intellectual disability, autism (Phelan et al., 1993; Phelan and McDermid, 2011; Kolevzon et al., 2014) Bipolar disorder and Psychosis symptom presentations (Kolevzon et al., 2019; Vogels et al., 2021) Sensory stimulating behaviours (Phelan and McDermid, 2011) and sensory reactivity (Tavassoli et al., 2021) ↓ Pain perception (Phelan and McDermid, 2011) ↑ Sleep problems (Bro et al., 2017; Smith-Hicks et al., 2021) ↓ Recognition memory and altered attentional engagement (Guillory et al., 2021)
Brain phenotype	↓ Glutamate receptor expression and synapse number in forebrain neurons (Shcheglovitov et al., 2013) ↓ Dendritic outgrowth in cortical neurons (Kathuria et al., 2018) ↓ Excitatory synapse number, ↓dendritic spines and outgrowth in cortical neurons transplanted into mice prefrontal cortex (Chiola et al., 2021) Hyperpolarisation channelopathy phenotype in human ESC cortical neuron (Yi et al., 2016)	Cortico-striatal hyperactivity (Peixoto et al., 2016) Cortico-amygdala hyperactivity (Kim et al., 2022) ↓ Hippocampal NMDA/AMPA excitatory ratio (Kouser et al., 2013) ↓ Excitatory transmission in hippocampal CA1, ↓ inhibitory synaptic events in medial prefrontal cortex (Lee et al., 2015) ↑ Spontaneous and stimulus-evoked activity of excitatory neurons in somatosensory cortex and ↓ interneuron activity (Chen et al., 2020) MRS: ↓glutamate concentration in striatum sMRI: ↓ total brain (Ellegood et al., 2015; Schoen et al., 2019), ↓ hippocampal (Schoen et al., 2019), and prefrontal cortex volume (Pagani et al., 2019), and ↑ Increased striatum volume (Peça et al., 2011; Wang X. et al., 2016) DTI: ↓ white matter microstructural architecture in external capsule (Golden et al., 2021), fronto-occipital association fibers and motor cortex-entorhinal cortex tract (Jesse et al., 2020)	Epilepsy/seizures (Kolevzon et al., 2019) EEG: ↓ VEP amplitude (Siper et al., 2022) EEG: Preserved auditory neural response and habituation; auditory processing variability linked to genetic subtype(Isenstein et al., 2022) EEG: ↑ phase amplitude coupling phase bias in posterior brain regions (Mariscal et al., 2021) fMRI: ↑activity for communicative sounds in the right superior temporal gyrus (Wang A. T. et al., 2016) sMRI: ↓ total brain vol, basal ganglia, and cerebellum in some studies DTI: ↓ corpus callosum white matter, and overall white matter thinning and altered uncinate fasciculus, inferior fronto-occipital fasciculus, and corticostriatal tract (Jesse et al., 2020)

Discrepancies also exist between preclinical animal models and human NRXN1 deletion and PMD clinical phenotype studies. Marked aggression in male Nrxn1 mouse models has not been reported commonly in NRXN1 deletion carriers in clinical studies. Anxiety is reported in Shank3 animal models but is not a common clinical feature in human studies. Hyperactivity and sleep disturbance reported in some human studies has not been reported in Nrxn1 animal models. Sex-differences in behavioural features of Nrxn1 murine models have not to date been reported in human studies. There have been no published clinical studies to date of learning and cognition in NRXN1 deletion carriers. To date, there is a gap in the NRXN1 deletion clinical literature of published neuroimaging and electrophysiology studies in carriers preventing comparisons with preclinical Nrxn1 models.

Inconsistencies between preclinical animal model and hiPSC and ESC studies have also been observed. iPSC derived from autistic people with NRXN1 deletion show hyperexcitability while in contrast human ESC-derived cortical neurons show reduced synaptic transmission and neurotransmitter release. These differences could potentially be related to differences in the genetic backgrounds of patient derived compared with ESC models. As discussed below, patient derived models may be enriched for multiple genetic risk variants due to clinical ascertainment, requiring careful consideration of appropriate controls for these studies in future. In animal models both reduced hippocampal synaptic strength and no difference in synaptic neurotransmission have been reported in Nrxn1 animal models. The discrepancies observed in preclinical models may be explained by methodological or species differences, but also by comorbidities in people, which are more rule than exception. Comorbidities can be due to additional genetic as well as environmental factors. Because it is challenging, if possible at all, to model multiple factors in a translational manner, comorbidities are seldom investigated in animal studies. Models are relatively simple per definition, to function as model and to be able to study a specific biological process. This is simultaneously the limitation of models.

Studies of brain phenotypes in PMD human and Shank3 animal model studies show parallel findings of reduced brain volume and altered white matter phenotypes, potentially suggesting a cross-species structural biomarker. Human and animal electrophysiology studies both indicate E/I imbalance, however, in contrast there were no observed differences striatum glutamate observed in a preclinical Shank3 animal model, which were reported in PMD.

Multiple studies in both preclinical studies of iPSC-neurons and rodent models of autism have shown hyperexcitability, strongly implicating Shank3 in E/I imbalance (Shcheglovitov et al., 2013; Yi et al., 2016; Chiola et al., 2021) and indicating the potential for successful cross-species phenotyping. While the translation of findings from *in vitro* to *in vivo* or vice versa has been demonstrated in autism preclinical models, the translation to clinic has not yet been achieved for autism related synaptopathies.

### 5.2. Gaps in our knowledge

The NRXN-NLGN-SHANK pathway is arguably one of the more complex molecular synaptic pathways. This is primarily due to the splice isoform diversity of NRXNs, the variable effects that SHANK mutations have on circuit level dysfunction and how each of these components contribute to synaptogenesis, synapse maturation, and E/I imbalance. However, substantial gaps in our understanding of how the roles of these molecules change in co-ordination with each other throughout the brain and across neurodevelopment at higher spatio-temporal resolution. Specifically, it is unclear whether NRXN-NLGN-SHANK interactions operate as a trans-synaptic macromolecular complex, and if so, how high- and low-penetrance autism associated mutations impact on function and clinical outcomes.

E/I imbalance is a common component of many conditions that emerge in atypical neurodevelopment or degeneration of neuronal subpopulations, e.g., schizophrenia (Gao and Penzes, 2015), epilepsy (Staley, 2015), ADHD (Rivero et al., 2015), and Alzheimer’s disease (Varela et al., 2019). The specificity of the phenotype in autism is unclear, indeed E/I imbalance may not be causal but could be a consequence of the interaction of genetic and cellular mechanisms that disrupt neural circuitry. A transdiagnostic role for E/I imbalance in neuropsychiatric conditions could represent an opportunity to repurpose drugs that modulate E/I balance in the context of autism (Ghatak et al., 2020). Ongoing gaps in our understanding of the underlying molecular mechanisms contributing to autism are a barrier to the translation of the results of preclinical studies to the clinical. Nevertheless, the positive evidence discovered thus far means that translating such complex molecular mechanisms into clinical treatments for autism-associated synaptopathies remains a convoluted goal for autism research. Research investigating multilevel brain and neurocognitive markers of NRXN1 deletions and PMD aligned to preclinical models is one of the ongoing aims of the AIMS-2-TRAILS consortium (Cooke et al., 2022), which is more broadly focused on understanding the heterogeneity and developmental trajectories of autism, as well as identifying biomarkers and testing efficacy of new treatments.

### 5.3. Challenges associated with human clinical heterogeneity and genetics

Genetic heterogeneity, discussed above, of the typical genetic mutations that contribute to NRXN1 deletions and PMD is a challenge to investigating genotype–phenotype associations in clinical studies. Genetic heterogeneity may partly explain differences in penetrance that are observed in PMD and NRXN1 deletion. The variable and incomplete penetrance of NRXN1 deletions remains to be explained but is likely related to gene–environment interaction or the influence of other common or rare genetic factors (Dinneen et al., 2022), further complicating cross-species comparisons. Evidence for the contribution of additional genetic variants to phenotype has previously been shown in another neurodevelopmental-associated CNV, 16p12.1 deletion, such that probands had a greater number of additional genetic variants compared to parent carriers of the deletion (Pizzo et al., 2019). Interestingly, the additional variants identified in probands were mainly brain expressed genes which may have contributed to the clinical and cognitive phenotypes observed in probands compared to the milder phenotype in parent carriers. Further, it is possible that both common and rare genetic variation contribute to autism and may influence phenotypic outcomes (Gaugler et al., 2014; Dinneen et al., 2022). Family-based designs, including parent and sibling carriers and non-carriers may prove invaluable for understanding phenotypic heterogeneity within synaptopathies such as NRXN1 deletion which is inherited more frequently than PMD.

While links between clinical phenotype and deletion size and location have been reported for both NRXN1 deletion (Cosemans et al., 2020) and PMD (Tabet et al., 2017), adequately powered investigations of associations between different subtypes of genetic deletions are limited by the availability of adequately powered and well-characterised cohorts. Large scale international collaborative efforts are imperative, such as the Developmental Synapatophies Consortium, which is focused on deep phenotyping, brain electrophysiological markers and developmental trajectories of three autism-related genetic conditions including PMD, Tuberous Sclerosis Complex and PTEN Hamartoma Tumor Syndrome (Levy et al., 2022), and the newly established R2D2-MH consortium which aims to combine samples across multiple cohorts to investigate genetic and environmental risk and resilience factors for mental health outcomes in neurodevelopmental conditions. Investigating NRXN1 deletions One goal is to incorporate a family-based design to better understand genetic background impacts on the heterogeneity in clinical and behavioural outcomes in NRXN1 deletions, and a second goal is to generate brain organoids to examine neurobiology and identify markers of resilience in people with and without rare genetic variants. These studies will inform the wide heterogeneity observed in neurodevelopmental-associated genetic variants such as NRXN1 deletion.^1^

The significant genetic heterogeneity in clinical studies hampers cross-species comparisons between preclinical and clinical since pre-clinical studies, in particular many murine models tend to focus on complete gene knock-outs. iPSC models derived from individuals with PMS or NRXN1 deletions have been investigated, but these require careful consideration of the background genetic effects (Schnabel et al., 2012) and control isogenic lines. Introducing structural variants into murine or iPSC models is enabled by Clustered Regularly Interspaced Short Palindromic Repeats (CRISPR) technology which should allow for the creation of models based on human specific mutations.

Related to this, clinical study design contributes to the difficulty in estimating the penetrance of NRXN1 deletions due to ascertainment biases. Clinical studies ascertain individuals with NRXN1 deletions identified due to the presence of a neurodevelopmental condition and are potentially enriched for deleterious variants. Population based studies in contrast are biased and under ascertain individuals with neurodevelopmental and neuropsychiatric conditions (Bycroft et al., 2018). Family based designs (Chawner et al., 2019; Nudel et al., 2022) offer an alternative to investigate penetrance in the context of the familial genetic background and include parent carriers who were not ascertained in childhood. The rarity of synaptopathies complicates the selection of reliable age and IQ appropriate measures of cognition and behaviour due to the necessity for liberal inclusion criteria. This type of measurement challenge differs from highly controlled preclinical studies which can focus studies in genetically heterogeneous models at specific developmental epochs. While overlaps in behavioural traits and some brain phenotypes have been observed between animal and human studies, recapitulating this wide genetic and clinical heterogeneity in animal models remains a challenge to cross-species translation.

### 5.4. Challenges associated with iPSC models and barriers to translation

Modelling neuronal circuitry is highly complex and is a primary challenge in the translation of outcomes from of hiPSC-neuron studies to animal models of synaptopathies. Two dimensional (2D) iPSC-neuron culture can reveal the molecular basis of neuronal circuitry but cannot feasibly replicate it’s intricacies (Chiola et al., 2022). Three dimensional (3D) iPSC-models, such as organoids and assembloids, more closely represent *in vivo* circuitry but they lack the coordination and organisation necessary to mimic accurate circuitry-level communication without cortical implantation (Revah et al., 2022). However, this approach with not without its technical challenges. For example, specialised approaches could be required for being able to image circuitry within organoids without 3D integrity (Adhya et al., 2021a).

Moreover, the technology to record network-level activity from multiple disparate cells simultaneously in the same whole intact organoid has yet to be fully realised. For example, it may be possible to develop a 3D structure of microneedle electrodes within which an organoid is growing to record neuron–neuron communication throughout the organoid (Choi et al., 2021), rather than a handful of cells on the surface of a sliced organoid (Sharf et al., 2022). It is possible to reliably quantify circuitry using viral labelling and retrograde tracing (Miura et al., 2022), low-throughput methods such as patch-clamp electrophysiology (Avazzadeh et al., 2021; Rosholm et al., 2022), or high-throughput methods such as multi-electrode arrays (MEAs; Alsaqati et al., 2020). MEAs have been deployed to investigate gene mutations associated with monogenic forms of autism in hiPSC-neurons such as *CNTN5, EHMT2, KCNQ2, ATRX,* and *SCN2A* (Deneault et al., 2018; Deneault et al., 2019). Such investigations can characterize the electrophysiological properties of human neurons with autism-associated gene mutations *in vitro* and can also validate the findings of *in vivo* electrophysiology or vice versa. Highly novel *in-vivo* high-throughput technology, e.g., Neuropixels probe, provides the capacity to record neuronal communication from >6,000 neurons simultaneously from multiple cortical and subcortical layers is groundbreaking although currently beyond the reach of most studies (Steinmetz et al., 2021). It has yet to be deployed in a disease model organism, but emerging biotechnology will likely provide a more accessible bridge between *in vivo* and *in vitro* electrophysiology over time.

IPSCs display variability in their capacity for differentiation and maturation which is an additional constraint. Gene editing technologies such as CRISPR associated (Cas) systems-9 in iPSC lines have proven instrumental in producing disease-associated lines with a distinct genetic background, thereby bridging a crucial gap in research. Using isogenic iPSC lines derived from well-characterized control iPSCs can bypass the heterogeneity and genetic differences of iPSCs. Moreover, another critical consideration to take into account with this approach, is the genetic background of the iPSC-line used in such studies. For example, in a recent study where heterozygous mutations in three autism-associated genes were engineered in multiple lines; the magnitude of phenotype differed between genetic background (Paulsen et al., 2022). This strongly indicates that other factors within the genome were able to impact the extent to which these genes impacted the observed phenotype. Nevertheless, CRISPR screens are already proving a significant boon to identifying new networks of genes implicated in neurodegenerative disease pathogenesis (Cooper et al., 2022). A related CRISPR-based approach was most recently deployed to investigate similarly disrupted gene networks in rodent models of autism (Jin et al., 2020), and a similar approach has been performed using iPSCs (Cederquist et al., 2020). Thus, it is likely only a matter of time before more CRISPR screen-based approaches are applied to human models of neurodevelopmental conditions (Townsley et al., 2020; Seah et al., 2023). To date, no study has simultaneously edited NRXN, NLGN, and Shank3 genes in the same system. Potential challenges here are off target effects (more pronounced in iPSCs due to their epigenetic memory and genetic background (Kim et al., 2010, Rouhani et al., 2014)), unclear direct causality, increased lethality, and increased burden of validating multiple edits. The approach has shown that schizophrenia-associated common risk variants of small effect interact synergistically to converge on synaptic perturbation rather than one or a handful of mutations directly leading to synaptic dysfunction (Schrode et al., 2019).

### 5.5. Methodological barriers and potential solutions to developing cross species phenotypes

There is an enormous need for the development of cross-species methods to advance, translation, from preclinical to clinical studies. Behaviourally, mice and rats can be trained on neurocognitive tasks using (touchscreen-based) comparable to those used in humans (Palmer et al., 2021). These approaches are conceptually and technically similar to the Cambridge Neuropsychological Test Automated Battery (CANTAB) and NIH Toolbox Cognition Battery developed for testing human cognition. For example, mice lacking the *Dlg2* gene (*Dlg2^−/−^*) and humans with *DLG2* CNV deletions (Dlg2 is a postsynaptic scaffold protein) displayed a comparable impairment in the object-location paired-associates task (Nithianantharajah et al., 2015). Shank3B heterozygous knockout mice were found to be slow to reach criterion in a visual discrimination task (Silverman et al., 2013; Copping et al., 2017). Even still, methodological considerations mean that these are not entirely comparable between species. Cognitive testing in humans is typically completed in 10–30 min per task while testing in rodent is conducted over several months and is impacted by additional factors. Rodents often need to be food restricted, and consequently socially isolated in the homecage, in order to motivate them to complete the tasks. These factors induce stress and physiological changes interfering with cognitive performance. Paradigms investigating sensory (gating) and stereotypies are less influenced by environmental factors compared with anxiety, social behaviour and repetitive behaviour.

The artificial environment of animal housing is often overlooked as a factor in animal behaviour studies. A recent meta-analysis showed that housing in shoe box-sized cages causes increased anxiety- and depression-like behaviour compared to more spacious and enriched housing. This may provide reproducible results, but they are of low ecologically validity. Potential solutions to improve the sensitivities of behavioural assays include advances in homecage monitoring combined with machine-learning methods to accurately measure detailed behaviours in mice over 24 h periods (Clapcote, 2022). Related to this, clean laboratories are associated with immature immune responses in mice and it is essential to house the animals in dirty environments, or to expose them to wild or pet mice, in order for natural microbiota and pathogens to develop in various sites in the body, and thereby more translational immune responses (Rosshart et al., 2019).

MRI is non-invasive, and comparable data approaches can be applied to humans and animals. For example, three large-scale brain networks were recently characterized by conserved spatio-temporal elements in mice and humans. MRS translational studies measuring glutamate and GABA metabolites have shown similar results in rodents and humans in the context of autism (Horder et al., 2018). PET is another potential cross species measure (Carey et al., 2022), however has not been implemented in PMD or NRXN1 deletion human clinical studies. EEG is another method of brain electrical activity, that can be used as a proxy measure for E/I balance (Ahmad et al., 2022), and has been implemented in both human and animals (Sohal and Rubenstein, 2019; Ahmad et al., 2022). Accessibility and feasibility of certain methodologies in clinical studies, such as MRI and EEG, prevent the advancement of translational research in autism and associated rare syndromes. Intellectual disability and autistic traits are common features and create sensory and behavioural barriers to administration. Various preparation strategies are used to improve accessibility and representation of the clinical spectrum, such as familiarization, desensitization, distraction techniques, communication strategies, behavioural strategies, coping strategies, and breathing techniques (Hallowell et al., 2008; Webb et al., 2015; Nordahl et al., 2016; DiStefano et al., 2019; Stogiannos et al., 2022). The reported success of these strategies is between 94 and 100%, but with a great deal of time and clinical expertise invested. Future work to address accessibility of neuroimaging techniques and different technologies will serve to improve the translational currency. Taken together, there are prospects to enhance the translational value of animal studies (Cait et al., 2022).

### 5.6. Animal and iPSC models as a platform to test interventions

Multiple therapeutic strategies have been proposed and tested in animal models, which might lead to translational implications for autism, NRXN1 deletion and/or PMD. For example, based on the molecular and physiological abnormalities in SHANK3-deficient animals, possible treatment options ranging from mechanisms targeting glutamatergic neurotransmission to epigenetic regulation of gene expression have been investigated (Wang X. et al., 2016; Vicidomini et al., 2017; Guo et al., 2019; Rhine et al., 2019). Although some treatments show promising results, a comprehensive screening for side effects has only rarely been reported. Effectiveness of treatments is also impacted by timepoint of intervention and type of compound, and to date most of the pharmaceutical compounds have not been translated into clinical routine, although there has been preliminary evidence of beneficial effects for PMD (Delling and Boeckers, 2021). Success in translating preclinical findings to clinic may potentially be increased by examining the efficacy of therapeutics and response to treatment in animal models that are more comparable to human clinical genetic variants of *NRXN1* or *SHANK3* mutations (i.e., induced with human mutations).

IPSC models have previously been shown to advance precision medicine in ultrarare pathogenic mutations (Sequiera et al., 2022), highlighting the potential utility of iPSC in high-throughput screening to identify the most effective treatment for an individual. This approach is potentially invaluable for both drug discovery and screening as it would help to improve treatment choices and care pathways for people, and reduce the effort of trialing multiple treatments that may be either ineffective or may lead to other adverse effects.

### 5.7. Future directions and conclusion

In this review we identified the challenges and opportunities for translation from preclinical to clinical research for synaptopathies. Significant domain specific advances in technologies and approaches are likely to progress our efforts towards translation. These include for example the development of technologies to record neuronal activity in 3-D brain organoids and the application of CRISPR to generate cell models with mutations reflecting those identified in autistic individuals. In animal models, it is increasingly feasible to implement cognitive batteries that are aligned with those used in humans. Challenges also need to be addressed, however. Animal preclinical model research would benefit from more ecologically valid environment when testing behaviour and cognition. More naturalistic conditions are imperative to understand how behaviour is truly influenced by genetic variants and environmental factors.

In the context of clinical research, significant heterogeneity exists even in rare syndrome presentations, and this is likely in part due to the background effects of common and other rare variants. Better characterisation at the genetic level using whole genome and exome sequencing will be necessary to fully understand the genetic architecture even for rare syndromes. Conducting these studies in a family-based design will also be a way to investigate pleiotropy and variable penetrance that is observed. Traditionally, neurobiology and genomics research has not significantly considered the role of environmental risk factors and the social determinants of health to aid the interpretation of the effects of genetic factors. Whether these can be modelled across the translational spectrum remains to be examined. We also highlighted the differences in developmental trajectories and sex/gender observed in synaptopathies in human and animal models which will necessitate approaches that use multiple modalities across critical periods of development and in both sexes.

One of the most obvious findings of the review is the high levels of variability in the approaches and models and the lack of comparability across different models. For example, since E/I balance is implicated there is future potential to align studies using neuroimaging and electrophysiological measures which may have potential as cross-species biomarkers. The limited number of neuroimaging studies to date, especially human studies of PMD and NRXN1 deletion, makes it difficult to make inferences about brain functional and structural development or identify the most robust brain biomarker. Cross-species studies will be important for establishing biomarkers to measure treatment efficacy. Finally, there is a growing need for increased dialogue between clinical and preclinical researchers to prioritise specific domains and phenotypes that have tractability across the translational spectrum.

## Author contributions

LG, DS, EL, CM, JC, and SS were responsible for the initial review article concept. CM, JC, NG, AR-O, SA, JH, JG, and CF contributed to the writing of the manuscript. LG and DS provided extensive input and critical revisions of the manuscript. All authors contributed to the article and approved the submitted version.

## Funding

This work was supported by the Innovative Medicines Initiative 2 Joint Undertaking under grant agreement no. 777394 for the project AIMS-2-TRIALS. This Joint Undertaking receives support from the European Union’s Horizon 2020 Research and Innovation Programme and EFPIA and AUTISM SPEAKS, Autistica, SFARI.

## Conflict of interest

The authors declare that the research was conducted in the absence of any commercial or financial relationships that could be construed as a potential conflict of interest.

## Publisher’s note

All claims expressed in this article are solely those of the authors and do not necessarily represent those of their affiliated organizations, or those of the publisher, the editors and the reviewers. Any product that may be evaluated in this article, or claim that may be made by its manufacturer, is not guaranteed or endorsed by the publisher.

## Footnotes

^1^http://r2d2-mh.eu/
